# *IFNL4* genotype influences the rate of HIV-1 seroconversion in men who have sex with men

**DOI:** 10.1080/21505594.2022.2066612

**Published:** 2022-04-28

**Authors:** Giovanna Meza, Fátima Galián, Claudia Jaimes-Bernal, Francisco J. Márquez, Faruk Sinangil, Carolina Scagnolari, Luis Miguel Real, Donald Forthal, Antonio Caruz

**Affiliations:** a Departamento de Biología Experimental, Unidad de Inmunogenetica, Universidad de Jaén, Jaén, Spain; b Universidad de Ciencias Aplicadas y Ambientales, Facultad de Ciencia y Tecnología, Bogotá, Colombia; c Universidad de Boyaca, Facultad de Ciencias de la Salud, Tunja, Colombia; d Global Solutions for Infectious Diseases, Lafayette, CA, USA; e Department of Molecular Medicine, Laboratory of Virology, Institut Pasteur Italia, SApienza University of Rome, Rome, Italy; f de Enfermedades Infecciosas y Microbiología Clínica, Hospital Universitario de Valme, Sevilla, Spain; g Inmunología, Universidad de MálagaDepartamento de Especialidades Quirúrgicas, Bioquímica e , Málaga Spain; h Division of Infectious Diseases, Department of Medicine, University of California, Irvine School of Medicine, Irvine, CA, USA

**Keywords:** HIV-1, HIV exposed seronegative, *HESN*, *IFNL4*, IL28B

## Abstract

Individuals lacking interferon lambda 4 (IFNL4) protein due to a common null mutation (rs368234815) in the *IFNL4* gene display higher resistance against several infections. The influence of IFNL4 on HIV-1 infection is still under discussion and conflicting results have been reported. This study intended to corroborate or refute the association of the null allele of *IFNL4* and HIV-1 predisposition in a cohort of men who have sex with men (MSM). *IFNL4* null genotype was assessed on 619 HIV-1-seronegative MSM who were followed for 36 months during a trial of a prophylactic vaccine against HIV-1. Of those, 257 individuals seroconverted during this period. A logistic regression model was constructed including demographic and *IFNL4* genotype. In addition, a meta-analysis using data from the current study and other European populations was conducted. The null *IFNL4* genotypes were correlated with lower HIV-1 seroconversion (Adjusted OR = 0.4 [95%CI: 0.2–0.8], P = 0.008) and longer time to seroconversion (889 vs. 938 days, P= 0.01). These results were validated by a meta-analysis incorporating data from other European populations and the result yielded a significant association of the *IFNL4* null genotype under a dominant model with a lower probability of HIV-1 infection (OR=0.4 [95% CI: 0.3-0.6]; P= 1.3 x 10E-5).

## Introduction

Genome-Wide Association Studies (GWAS) identified genetic variations upstream of *IFNL3* (IL28B) that are effective biomarkers for predicting spontaneous and interferon-alpha/ribavirin treatment-dependent cures of Hepatitis C virus infection [1,2]. The most significant SNP associated with these phenotypes was rs12979860 found upstream of *IFNL3*. Later, the functional variant was located in a new gene close to *IFNL3*, named *IFNL4* [3]. This variant (rs368234815) is an insertion-deletion polymorphism (INDEL) with a functional *IFNL4* coding allele (G) and a null allele (TT) that gives rise to a frameshift mutation and no IFNL4 protein. In contrast to the functional variant, rs12979860 is located in an intron, both variants are separated apart by 418 bp. INFL4 shows a potent antiviral activity *in vitro* [3], so it is perplexing that the deficit of this protein can increase the resistance to several infectious diseases.

Whether the genotype of *IFNL4* modifies the immune status of people living with HIV remains controversial, with conflicting observations reported [4,5]. G-*IFNL4* genotype (functional variant) is correlated with lower CD4+ lymphocytes in treatment-naïve and asymptomatic individuals [6], normalization of the CD4+/CD8+ lymphocyte ratio with higher proportions of naïve CD8+ *T*-cells and lower CD4+ effector memory cells after virological suppression [7]. On the contrary, the *IFNL4* genotype was not associated with failure to restore CD4 *T*-cell levels even with prolonged virological suppression [8]. Additionally, the G-*IFNL4* genotype was found to be associated with a higher prevalence of cytomegalovirus retinitis [9], tuberculosis and *Pneumocystis* pneumonia [6].

Martin et al [10]. examined the influence of *INFL4* on susceptibility in a cohort of HIV-1 positive vs. highly exposed uninfected from the United States, including individuals of European and African ancestry. They found no association with protection in highly exposed seronegative individuals. Similar results were found in a North African population [11]. However, these studies did not genotype the functional INDEL located in the open reading frame of the *IFNL4* but another SNP in the first intron. This fact has important statistical consequences since both variants are in complete linkage disequilibrium in Europeans and Asians but not in individuals of African ancestry [3]. The null allele of *IFNL4* was found to be highly protective against HIV-1 infection in intravenous drug users from Spain [12]and Estonia [13]. Moreover, Jaimes-Bernal et al [14]. found the same trend in highly exposed seronegative individuals at risk of infection through heterosexual contact with HIV-1-infected partners. Due to the previous contradictory observations, we intended to shed light on the association between the *IFNL4* polymorphism and sexually transmitted HIV-1 infection in MSM with known risk factors and the date of seroconversion. Additionally, *IFNL4* and HIV-1 susceptibility data from the current and from previously reported studies in European populations were all combined in a meta-analysis.

## Patients and methods

### Study population

We genotyped healthy MSM of European ancestry from the United States (VAX004 cohort). All of them were volunteers participating in a placebo-controlled phase 3 trial of a prophylactic vaccine against HIV-1 infection (Vax004 trial, Clinical-Trials.gov Identifier: NCT00002441) [15]. The vaccine candidate was based on two recombinant gp120 HIV-1 envelope proteins (MN and GNE8, AIDSVAX B/B; VaxGen) adsorbed onto 600 mg of alum. Individuals were eligible for entering the trial if they were not intravenous drug users, had anal intercourse during the preceding 12 months and not have a monogamous sexual relationship with an HIV-1 seronegative partner [15,16].

The vaccine did not demonstrate a protective effect: infection rates were 6.7% in vaccinees versus 7.0% in placebo recipients; vaccine efficacy was 6% (95% confidence interval, −17% to 24%). There were no significant differences in viral loads, initiation of antiretroviral therapy or infecting HIV-1 strains between treatment arms [15,16]. In this study, we included a random sample of volunteers with DNA samples available that were followed up 1200 days after vaccine or placebo administration.

The volunteers reported different sexual behavior at the study baseline and were classified according to the number of risk factors associated with HIV-1 seroconversion as high (risk score ≥2) or low risk (risk score <2) [15,16]. The reported factors included unprotected receptive anal sex with an HIV-1-infected male partner, unprotected insertive anal sex with an HIV-1-infected male partner, unprotected receptive anal sex with an HIV-1-uninfected male partner, five or fewer acts of unprotected receptive anal sex with a male partner of unknown HIV-1 status, 10 or fewer sex partners, anal herpes, hepatitis A, use of poppers and amphetamines.

### Genotyping and biostatistics

DNA extraction and genotyping were performed as previously described [14]. Pearson chi-square tests were used to compare categorical variables in two groups of individuals, Mann-Whitney U tests (data not normally distributed) were used for quantitative variables. Detection of independent risk factors related to HIV-1 seroconversion with adjusted *P*-values and OR were done by logistic regression models that include parameters with a univariate *P*-value <0.05. Estimated survival probabilities were calculated using the Kaplan-Meier method, and survival curves were compared by the log-rank test. Parameters associated with the time (days) from baseline to HIV infection were come into in a Cox regression to calculate adjusted *P*-values and hazard odds ratios. Calculations were performed using SPSS 21 software (IBM Corporation, NY, USA).

Hardy–Weinberg equilibrium and the different genetic association models (dominant, recesive) were calculated employing an online tool (https://ihg.gsf.de/cgi-bin/hw/hwa1.pl) [17] and the PLINK software [18]. Genotype data of rs12979860 or rs368234815 variants from current and previous populations of single European ancestry [12–14]were combined using a fixed-effect meta-analysis. Meta-analysis OR and 95%CI were calculated and represented with the online tool *MetaGenyo* developed at the “Centro Pfizer–Universidad de Granada–Junta de Andalucía de Genómica e Investigación Oncológica”, Granada, Spain (https://metagenyo.genyo.es/) [19].

### Ethics

This study was approved by the Institutional Review Board of the Province of Jaen, Junta de Andalucia (GEN-VIH/0646-*N*-20 version 1 of 9 March 2020 and Protocol “Identificación de factores genéticos de Resistencia innata a la infección por VIH-1” of 26 July 2018) and was designed and performed according to the principles of the Helsinki Declaration. All volunteers provided written informed consent to participate in this study.

## Results

Six hundred nineteen individuals were included. Three hundred and seventy-three (61%) had been treated with the recombinant glycoprotein 120 vaccine and 327 (52.8%) had a risk score for HIV infection ≥2. During the follow-up, 257 (41.5%) individuals became HIV infected (Table 1).

**Table 1.** Parameters associated with HIV infection during the follow-up

**Variables**	**HIV-non-infected (*n*=362)**	**HIV-infected (*n*=257)**	**Univariate** **p**	**Multivariate** **p, AOR (95%CI)**
Risk score>2, n (%)	161 (44,5)	166 (64,6)	<0.001	<0.001, 2.42 (1.72–3.39)
Age, years*	36 (31–44)	35 (29–41)	0.004	<0.001, .97‡ (0.95–0.98)
INFL4 GG/GT/TT, n (%)	30/144/188 (8.3/39.8/51.9)	39/89/129 (15.2/34.6/50.2)	0.007†	0.007†, .49 (0.28–0.82)

OR, Odds ratio; CI, Confidence interval.

*Median (quartile 1- quartile 3).

‡For one unit increase.

†TT/TT+TT+GT vs. GG.

*INFL4* rs368234815 genotypes distributions in both volunteers that became HIV-1 seropositive or maintained as HIV-1 negative are shown in Table 1. The frequency of *INFL4* T allele carriers was significantly different between the HIV-1 seroconverters vs. HIV-1 negatives (91.7% vs 84.8%, p = 0.007; Odds Ratio (OR) = 0.50, 95% confidence interval (95%CI) = 0.30–0.84). The protection conferred by the *INFL4 T* allele in regards to the HIV-1 infection was independent of other factors associated with HIV-1 such as younger age and risk score ≥2 (Table 1). *IFNL4* rs368234815 genotypes showed a deviation from Hardy-Weinberg equilibrium in individuals that became HIV-1 infected (p = 0.0007) but not in those who remained uninfected (p = 0.743). Similar results were obtained when both vaccine and placebo-treated groups were separately analyzed (Table 2).

**Table 2.** Genotypic distribution of *INFL4* genotypes in all the cohort, vaccinees and placebo-treated subjects of the Vax004 clinical trial

**Treatment**	**HIV seroconversion**	**Genotypesa**		
		**G/G**	**G/TT**	**TT/TT**
All	**HIV-positive**, n (%) P ^H-^^W^ = 0.0007	39 (15.2)	89 (34.6)	129 (50.1)
	**HIV-negative**, n (%)* P ^H-^^W^ = 0.4	30 (8.2)	144 (39.7)	188 (51.9)
	OR (95% CI), *P*-value ^b^	0.52 (0.3–0.8), 0.007		
	Adjusted OR (95% CI) and *P*-value^c^	0.48 (0.2–0.8), P= 0.008		
Vaccinees		**Genotypesa**		
		**G/G**	**G/TT**	**TT/TT**
	**HIV-positive**, n (%) P ^H-^^W^ = p=0.01	28 (15.2)	67 (36.4)	89 (48.3)
	**HIV-negative**, n (%) P ^H-^^W^ = 0.39	16 (8.2)	88 (45.3)	90 (46.3)
	OR (95% CI), *P*-value ^b^	0.52 (0.2–0.9), 0.03		
	Adjusted OR (95% CI) and P-value^c^	.49 (0.2–0.9), P= 0.043		
Placebo		**Genotypesa**		
		**G/G**	**G/TT**	**TT/TT**
	**HIV-positive**, n (%) P ^H-^^W^ = 0.01	11 (15.1)	22 (30.0)	40 (54.0)
	**HIV-negative**, n (%) P ^H-^^W^ = 0.14	14 (8.3)	56 (33.3)	98 (58.3)
	OR (95% CI), P-value ^b^	0.5 (0.2–1.2), 0.1		
	Adjusted OR (95% CI), and P-value^c^	0.45 (0.18–1.07), P= 0.07		

OR: Odds Ratio. CI: Confidence interval. ^H-^^W^ Test for deviation from Hardy-Weinberg equilibrium.

Data are genotype counts (%).

Univariate logistic regression.

Adjusted OR and adjusted P values were calculated using a multivariable logistic regression model including *IFNL4* genotype, age, risk of HIV-1 infection according to baseline sexual behavior and vaccination status.

**Figure 1.**
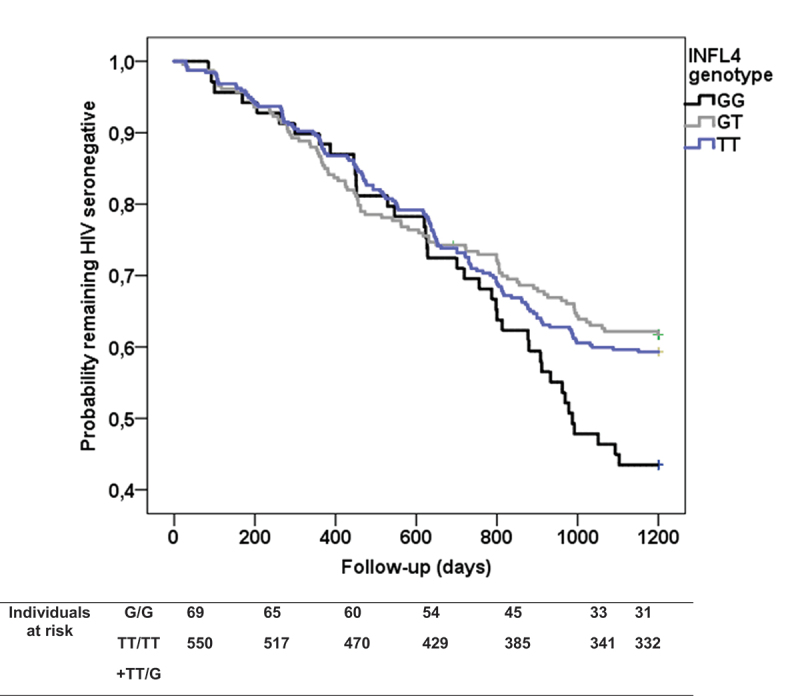
Kaplan-Meier plot of rate of HIV-1 seroconversion according to IFNL4 genotypes. Survival curves were compared under a dominant model for rs368234815 polymorphism (TT+GT vs. GG) by the log-rank test (P=0.01). G is the functional IFNL4 allele.

In addition, the *IFNL4* T carriers presented a longer time to HIV-1 seroconversion compared to the G/G genotype (938 vs. 889 days) (Figure 1). Again, this effect was independent of other factors also associated with this end-point (Table 3).

**Table 3.** Predictors of the HIV-1 seroconversion

**Variables**	**Events, n (%)**	**Univariate** **p; HOR (95%CI)**	**Multivariate** **p; HOR (95%CI)**
**Baseline sexual risk behaviour** score≥2 score<2	166 (50,8) 91 (31.2)	<0.001; 1.86 (1.44–2.41)	<0.001; 1,93 (0.1.50–2.50)
**Age**, years	-	0.002; 0.098‡ (0.97–0.99)	<0.001; 0.97‡ (0.96–0.99)
***INFL4*** TT/TT+TT+GT carriers GG cariers	218 (39.6) 39 (69.0)	0.019; 0.67 (0.47–0.94)	0.028; .68 (0.48–0.96)

HOR, Hazard odds ratio; CI, Confidence interval.

‡For one unit increase.

**Figure 2.**
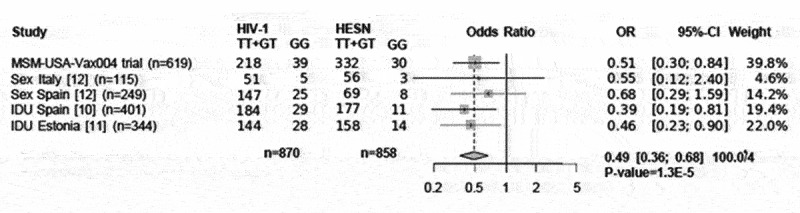
Forest plot of meta-analysis of the men who have sex with men (MSM-USA-Vax004 trial), serodiscordant couples (Sex Italy and Spain), and intravenous drug users (IDU) populations from Spain and Estonia (https://metagenyo.genyo.es/) [19]. Odds ratio (OR) and 95% confidence interval (95%-CI) and fixed-effect meta-analysis. P=1.3 x 10-5 for a dominant model of the protective allele (G/G vs.TT/TT+TT/G).

Available *IFNL4* genotype data from homogeneous populations of European ancestry (Spain, Italy and Estonia) at risk of HIV acquisition by IDU or sexual routes [12–14]were combined with the current data through a fixed-effects meta-analysis (n = 1728). The result yielded significant results with a dominant model for the protective TT allele (absence of IFNL4 protein) including the genotypes TT/TT+TT/GT vs.G/G (OR = 0.4 [95% CI, 0.3–0.6]; P = 1.3E–5; heterogeneity test P = 0.9, Figure 2).

## Discussion

Our results strongly suggest that individuals carrying the rs368234815 *IFNL4* T allele, which is a null allele of *IFNL4*, have less susceptibility to infection by HIV-1. Present data confirms in an MSM population at high risk of infection, the involvement of *IFNL4* in HIV-1 infection predisposition.

The role of IFNL4 in infectious disease susceptibility in general and HIV-1, in particular, is a topic of great interest due to the potential shared roles in innate immunity against several viral infections. Paradoxically, lacking IFNL4 is protective for relevant human viruses such as HCV [3]and SARS-COV-2 among others [20], whereas its presence is associated with reduced liver inflammation and cirrhosis in HCV-infected individuals [21,22]. The enigma goes beyond, as *IFNL4* functional gene orthologues are present in mammalian genomes from distantly related species to more close ones as chimpanzees, suggesting a positive role in the course of evolution [23]. The same applies to African populations where the frequency of the functional *IFNL4* allele is significantly higher compared to the rest of the world. Human populations outside of Africa show a clear positive evolutionary selection for a null *IFNL4* gene, and the biological basis for such selection is not established. The most plausible hypothesis is that ancient populations outside of Africa have been devastated by epidemics of pathogens that benefit from IFNL4-dependent signaling or aggravate the disease course due to lower inflammatory response mediated by IFNL4, thus driving a positive selection for *IFNL4* null allele carriers [14].

Based on previous observations [24], we propose that IFNL4 belongs to a negative feedback system, that occurs when the system´s output inhibits itself. This can be achieved by blocking the perception of new input signals or inhibiting the output response. Whereas positive feedback loops can lead to an exponential growth of the output signals, negative feedback promotes stability, settling to equilibrium and reducing the effects of distress [25]. That means that IFNL4 may induce a transient antiviral state but blocks further stimulation to avoid enhanced IFN-induced inflammation [26]. IFNL4 desensitizes the response to IFNA by inhibition of the JAK-STAT signaling pathway [27], causes refractoriness to stimulation with IFNL3 [24], and increases levels of inhibitors of the IFN response (SOCS1 and USP18) [24]. Moreover, the presence of the functional *IFNL4* gene leads to reduced liver inflammation and is associated with protection against cirrhosis in HCV chronic carriers [21]. In this context, functional IFNL4 possibly will induce a long-term insensitivity to interferon alpha, driving to weakened protection against HIV-1 infection.

This study has some limitations. First, we have genotyped only the INDEL rs368234815 that controls the production of IFNL4. However, another non-synonymous variant in the open reading frame of *IFNL4* causes a change from proline to serine at position 70 (rs117648444). The minor rs117648444-A allele (IFNL4-70-S variant) is less active compared with the more frequent IFNL4-70P variant. Importantly, only the rs368234815-G allele can harbor the functional rs117648444 polymorphism. The simultaneous genotyping of rs368234815 and rs117648444 may improve the association here described. However, the minor allele has a 12% frequency in European populations and due to this relatively low occurrence; its effect on our observed association may be marginal. Second, to have more statistical power, we have not taken into account the possible effect of the vaccine in our main results. However, the recombinant glycoprotein 120 vaccine demonstrated no effect in the prevention of HIV infection in the entire VAX004 cohort [15]. Third, this study was performed on a randomly selected sample of individuals belonging to the VAX004 cohort. Therefore, selection bias cannot be excluded. However, the results obtained are in accord with those previously published by us and others [12–14]. Finally, studies that tested rs12979860 in populations of African ancestry were not included in the meta-analysis. Two studies comprising African-American [10] and Moroccan [11] individuals were excluded in the meta-analysis since they tested the rs12979860 SNP located in the first intron instead of the functional INDEL rs368234815 located in the protein-coding region of *IFNL4*. Europeans and Asians show complete linkage disequilibrium for both genetic polymorphisms, that is not the case for it for African populations where both genetic markers are genetically independent.

In conclusion, this study (1) replicates in an independent population of MSM the relationship between *IFNL4* and innate resistance to HIV-1 and (2) establish the effect size of the IFNL4 null allele in the protection against HIV infection in European populations.

## Data Availability

Due to the nature of this research, participants of this study did not agree for their data to be shared publicly, so supporting data is not available. The data generated during the current study are available from the corresponding author on reasonable request.
